# Metabolism in atherosclerotic plaques: immunoregulatory mechanisms in the arterial wall

**DOI:** 10.1042/CS20201293

**Published:** 2022-03-29

**Authors:** Maria J. Forteza, Daniel F.J. Ketelhuth

**Affiliations:** 1 Cardiovascular Medicine Unit, Center for Molecular Medicine, Department of Medicine, Karolinska Institute, Karolinska University Hospital, Stockholm, Sweden; 2 Department of Cardiovascular and Renal Research, Institute of Molecular Medicine, University of Southern Denmark, Odense, Denmark

**Keywords:** atherosclerosis, cardiovascular disease, immunometabolism, immunomodulation, macrophages, T-cells

## Abstract

Over the last decade, there has been a growing interest to understand the link between metabolism and the immune response in the context of metabolic diseases but also beyond, giving then birth to a new field of research. Termed ‘immunometabolism’, this interdisciplinary field explores paradigms of both immunology and metabolism to provided unique insights into different disease pathogenic processes, and the identification of new potential therapeutic targets. Similar to other inflammatory conditions, the atherosclerotic inflammatory process in the artery has been associated with a local dysregulated metabolic response. Thus, recent studies show that metabolites are more than just fuels in their metabolic pathways, and they can act as modulators of vascular inflammation and atherosclerosis. In this review article, we describe the most common immunometabolic pathways characterised in innate and adaptive immune cells, and discuss how macrophages’ and T cells’ metabolism may influence phenotypic changes in the plaque. Moreover, we discuss the potential of targeting immunometabolism to prevent and treat cardiovascular diseases (CVDs).

## Introduction

Atherosclerosis is the main underlying cause of cardiovascular diseases (CVDs), such as myocardial infarction, stroke, and peripheral vascular disease, which lead to high mortality and morbidity worldwide [1]. Atherosclerosis is a multifactorial disorder linked to several other diseases and/or risk factors, including hyperlipidaemia, diabetes mellitus, hypertension, smoking, and sedentarism [2]. The atherosclerotic process starts very early in life, and fatty streaks, which are the first signs of disease, have been reported in human foetuses from hypercholesterolaemic mothers [3]. In most cases, atherosclerosis remains asymptomatic for decades. However, changes occur when significant reductions in blood flow caused by plaque growth and luminal stenosis or acute thrombotic obstruction induced by endothelial erosion or plaque rupture.

Currently, the foremost strategy to prevent atherosclerosis focuses on managing traditional risk factors, while the treatment of disease complications often relies on interventional and surgical procedures, such as percutaneous coronary intervention, stent implantation, and endarterectomy, all of which aim to re-establish blood flow. Despite recent progress, existing therapies are likely to reduce the incidence of heart disease by one-third at most [4]. In this dismal scenario, CVD remains a heavy burden on our society, and more than 17 million annual cardiovascular-related deaths are reported globally, which is approximately 31% of all-cause mortality [2]. Sadly, projections indicate that by 2030, nearly 24 million individuals will die of CVD yearly if disease care cannot be improved [5].

A large body of evidence points towards the immune system interacting with classic risk factors to drive vascular inflammation, which is the most important factor driving plaque formation and instability. In this context, there is high hope that lives can be saved with the use of immunoregulatory therapies [6,7]. Although human trials targeting inflammation in secondary prevention of CVD have been achieved, it is likely that a deeper understanding of the pathophysiological processes driven by immune cells in the arterial wall is still necessary for major breakthroughs. In this review, we summarise some recent developments in the field and discuss the role of immunometabolism in atherosclerotic plaques and its potential for modulating inflammation and improving cardiovascular medicine.

## The immune system and atherosclerosis: a brief overview

It is well recognised that atherosclerosis is a chronic inflammatory disease initiated by the retention and modification of low-density lipoprotein (LDL) in the intimal layer of large- and medium-sized arteries, which activates the endothelium and promotes immune cell infiltration [8]. Monocyte-derived and tissue-resident macrophages are the most common immune cell types found in growing plaques [9]. After dysregulated uptake of modified LDL [10], macrophages may become foam cells that are trapped in the vessel wall, eventually die and augment the local inflammatory process [11].

Primarily composed of smooth muscle cells (SMCs) and collagen, a fibrous cap is formed to stabilise the lesion and prevent prothrombotic molecules of the intima from contacting the bloodstream. However, the complex inflammatory process within the vascular wall can lead to high expression of matrix metalloproteinases (MMPs) and other mediators that can cause thinning and rupture of the plaque [12,13].

Not only macrophages but also T cells are abundant in plaques. The most common T-cell population, T helper (Th) 1 cells, has been proposed to accelerate disease, especially by secreting interferon γ (IFNγ), which acts in a proinflammatory and plaque-destabilising manner [6]. Hypercholesterolaemia has been associated with the expansion of distinct CD8+ T-cell types that can also influence disease [14]. In addition to inhibiting SMC proliferation and reducing their capacity to produce collagen, IFNγ can induce M1 macrophage polarisation, and the secretion of interleukin (IL) 1β (IL-1β), IL-12, and TNF, all key players in atherogenesis. The milieu of cytokines produced by immune and vascular cells can induce high expression levels of the costimulatory molecules CD80 and CD86 and major histocompatibility complex (MHC) class II (MHC-II), which creates a proinflammatory feed–forward loop leading to increased Th1 responses [15–17].

Counteracting the proinflammatory responses in the vascular wall, M2 polarised macrophages and regulatory T cells (Tregs) are thought to strongly counteract disease [6,18]. M2 macrophages secrete IL-10 and resolving lipid mediators that can limit immune activation [19], increase efferocytosis, and promote the resolution of vascular inflammation [20,21]. Tregs further support the latter processes by different mechanisms, including the inhibition of type I responses, through the secretion of TGFβ and IL-10, and contact-dependent cell-mediated effects, such as mediating cytotoxic T lymphocyte-associated protein 4 (CTLA4) interactions with CD80/CD86 and promoting M2 macrophage polarisation [22].

Both innate and adaptive immune responses can influence all stages of atherosclerosis [23]. Data from animal models indicate that modulating immune cell repertoires and their secreted mediators in the arterial wall, such as by targeting specific immune cells, inhibiting/blocking cytokines and costimulatory molecules, and using different vaccine and tolerisation protocols, can prevent atherosclerotic CVD [7,24]. The successful reduction in the number of cardiovascular events and deaths demonstrated in recent clinical trials using anti-IL1β monoclonal antibodies [Canakinuamb Anti-inflammatory Thrombosis Outcomes Study (CANTOS); ClinicalTrials.gov; NCT01327846] and colchicine [Low Dose Colchicine for secondary prevention of cardiovascular disease (LoDoCo2 Trial); UTN: U1111- 1139-8608] strongly suggests that immunomodulation could be a relevant therapeutic option to combat atherosclerotic CVD in humans, in addition to the management of classic risk factors [25–27]. Despite that, canakinumab has not been approved by the FDA for treatment of atherosclerotic CVD, due to some of its side-effects, namely increased risk of severe infections and sepsis. Another trial (Cardiovascular Inflammation Reduction Trial—CIRT) attempting to use a broad-spectrum anti-inflammatory agent, methotrexate, to prevent CVD showed neutral results [28]. Collectively, these trials show that there is a need for further understanding of the specific inflammatory pathways governing atherosclerosis to achieve the goal of improving cardiovascular medicine.

## Immunometabolism: metabolic regulation of macrophage and T-cell responses

Metabolism and the induction of immune responses are very closely linked. In addition to fueling the most basic immune cell functions, such as the synthesis of immune mediators such as cytokines and chemokines and the formation of new cell membranes and allowing proliferation, microenvironmental or systemic abundance of different nutrients, and their metabolism, can modulate immune cell differentiation and polarisation, as well as their function, and result in protective or deleterious responses. Thus, the growing field of immunometabolism research indicates that cellular metabolic pathways are not only housekeeping processes to maintain cell survival but also active modulators of disease processes that go beyond the spectrum of metabolic disorders, including cancer, infection, and autoimmune and autoinflammatory diseases [29]. In this section, we summarise major studies exploring the processes involved in the metabolic reprogramming of immune cells, especially the energy-related pathways, and the potential of targeting immunometabolism in macrophages and T cells to control inflammation.

Cellular metabolism to generate ATP involves a series of thermodynamically unfavourable reactions that, in addition to energy, can provide the building blocks needed for the synthesis of macromolecules. Energy metabolism is dynamically regulated in macrophages and can help these cells adapt to new functions. Glucose is the main source of ATP for most cell types, including macrophages and T cells. Once glucose crosses the plasma membrane and enters the cell, it is metabolised through two main metabolic pathways: glycolysis and the pentose phosphate pathway (PPP). Under normoxia, it is expected that glycolysis regulates the formation of pyruvate, which is then further metabolised through the TCA cycle in the mitochondria, involving a series of reactions called oxidative phosphorylation (OXPHOS), that generates 36 ATP molecules. In an anaerobic environment, it is expected that pyruvate metabolism will lead to increased lactate conversion and lower amounts but faster rates of ATP production (2 molecules).

In the 1920s, Otto Warburg observed that tumour cells have up-regulated glycolysis compared with surrounding normal tissue, with increased fermentation of pyruvate to lactate rather than oxidation in mitochondria, even in the presence of oxygen [30]. This peculiar form of energy metabolism, which is similar to anaerobic metabolism, has been termed ‘aerobic glycolysis’ and subsequently the ‘Warburg effect’. It has long been proposed that this characteristic metabolism may represent a selective adaptation associated with immune escape mechanisms of cancer cells. Recently, several groups demonstrated that immune challenge and leucocyte activation promote metabolic reprogramming that shifts metabolism from oxidative towards ‘Warburg metabolism’ [31,32]. Although aerobic glycolysis is a relatively inefficient way to generate ATP compared with OXPHOS, this method seems to be very important for quickly providing metabolic intermediates for key biosynthetic pathways that are needed during inflammation [33].

### The metabolic profiles of macrophages

In the steady state, quiescent M0 macrophages acquire energy by efficiently using the OXPHOS pathway [34]. In contrast, polarised macrophages (M1 and M2) seem to acquire metabolic characteristics of energy production that reflect distinct microenvironments [35]. At their extreme polarisation states, classical M1 macrophages, which can be induced by LPS and IFNγ, and nonclassical M2 macrophages, which can be induced by IL-4 and IL-13, exhibit very distinct metabolic profiles [36]. It should be highlighted that the terms M1 and M2 are clear oversimplifications of a vast repertoire of possible phenotypes that can be generated *in vivo* [37], and develop within an inflamed tissue, including atherosclerosis. Recent studies employing single-cell RNA sequencing (scRNAseq) and Cytometry by time of flight (CyTOF) techniques to atherosclerotic plaque-derived cell suspensions have revealed at least five major macrophage clusters infiltrating the artery of mouse models (three major clusters termed ‘resident-like’, ‘inflammatory’, and ‘TREM2 foamy’ macrophages, and two less abundant clusters termed ‘IFN-inducible’ and ‘cavity macrophages’), which were to some extent similar in human plaques [38–45]. Therefore, hereafter the terms M1 and M2 should be interpreted with certain degree of caution, or just as a general reference to pro- and anti-inflammatory phenotypes, respectively.

Downstream of Toll-like receptor 4 (TLR4) and IFNγ receptor (IFNR), NF-κB and Akt signalling trigger key metabolic alterations in macrophages, including increased glucose uptake [46–48] and the activation of the transcription factor hypoxia-inducible factor-1α (HIF1α), and increased glycolysis flow towards lactate formation due to up-regulation of the pyruvate dehydrogenase kinase 1 (PDK1) and lactate dehydrogenase A (LDHA) [49]. Notably, these changes are thought to be needed for M1 macrophages to effectively perform phagocytosis, produce reactive oxygen species (ROS), and secrete pro-inflammatory cytokines [50].

Parallel to glycolysis, in the cytosol, the PPP drives the generation of ribose-5-phosphate and nicotinamide adenine dinucleotide phosphate (NADPH). Ribose-5-phosphate is a precursor of nucleotides and amino acids, while NADPH is used by several enzymes in macrophages, including NADPH oxidase, which can influence the generation of ROS in macrophages [51].

Metabolic alterations in macrophages are not limited to glycolysis, and metabolite tracing experiments on M1 macrophages revealed that the TCA cycle is truncated, resulting in reduced succinate dehydrogenase (SDH) and isocitrate dehydrogenase (IDH) activities, consequently leading to intracellular overload of succinate and citrate, respectively [52]. Excess citrate shifting from the mitochondria to the cytosol increases acetyl-coenzyme A (CoA) production, which influences fatty acid synthesis and can boost the acetylation of gene promotors encoding inflammatory cytokines and chemokines [53]. In this context, ATP-citrate lyase (ACLY), which catalyses the transformation of citrate into acetyl-CoA and oxaloacetate, is induced by TLR ligation and has been implicated in the regulation of IL-1β, CXCL1, IL-6 and IL-12 production [54]. Accumulated succinate in the cytosol can stabilise HIF1α, independent of normoxic or hypoxic conditions, and influence the transcription of metabolic and inflammatory mediators [55]. Succinate can also influence inflammation through the succinylation of pyruvate kinase M2 (PKM2) and active signalling via G protein-coupled receptor-91 (GPR91); both mechanisms that have been associated with the regulation of IL-1β production [55,56].

In addition to glycolysis and the PPP, glucose can be metabolised via the hexosamine biosynthesis pathway (HBP), leading to the generation of uridine diphosphate N-acetylglucosamine (UDP-GlcNAc) [57]. Several studies have demonstrated that O-linked B-N-acetylglucosamine (O-GlcNAc) signalling promotes an inflammatory response in macrophages [58,59]. O-GlcNAcylation can affect the transduction and transcription of key proinflammatory proteins, including NF-κB, in macrophages [60]. However, in certain scenarios, such as during ischaemia and sepsis, O-GlcNAcylation could also confer anti-inflammatory properties on macrophages [61,62], indicating that further investigation is needed to fully understand the role of this pathway in immunity.

When glucose availability is limited, ATP may be generated through fatty acid oxidation (FAO). Macrophages are capable of taking up different forms of lipids, such as free fatty acids bound to albumin, as well as LDL, VLDL, HDL and modified lipoproteins, which are typically mediated through specific receptors, including scavenger receptors such as SRA and CD36 [63,64]. Intracellularly, free fatty acids reach the mitochondria, where FAO takes place, and lead to the production of acetyl-CoA, NADH and FADH2, which may enter the TCA cycle and the electron transport chain to produce ATP [65].

M2-polarised macrophages mainly promote anti-inflammatory responses and healing. In general, the metabolism of M2 macrophages is comparable with that of quiescent macrophages and is shifted towards OXPHOS and FAO [64]. This propensity of M2 macrophages to metabolise lipids is also associated with increased lipoprotein lipase (LPL) and CD36 expression, which facilitates the uptake and intracellular transport of fatty acids [66]. However, recent studies have suggested that metabolism in M2 macrophages could be more complex than expected, and it was shown that carnitine palmitoyl transferase 2 (CPT2), a gatekeeper of FAO, is dispensable for M2 macrophage polarisation [67], while glycolysis plays a key role in the early steps of M2 polarisation [68]. Of note, some of the discrepancies in the literature regarding the metabolic profiles of macrophages could be due to nonspecific effects of inhibitors, such as 2-deoxy-d-glucose (2-DG), which, despite being used as an inhibitor of glycolysis, can also influence OXPHOS [69].

Amino acids are also essential nutrients for the immune system. During inflammation, amino acid deficiency may result in impaired immune cell migration, proliferation, and effector functions. Thus, altered amino acid metabolism is likely to affect macrophage responses through the generation of bioactive catabolites that can act as signalling molecules on these cells [70].

Macrophages utilise glutamine at high rates and are dependent upon extracellular sources of this amino acid. Glutamine can feed the synthesis of other amino acids, nucleotides, and NADPH, and it constitutes a key energy source. Interestingly, macrophage glutaminolysis seems to depend on the extracellular abundance of this amino acid, which can affect proliferation and other critical macrophage functions, such as phagocytosis, RNA synthesis and IL-1β production [33,71]. Having high glutamine flux towards the TCA cycle is a major mechanism by which succinate synthesis is promoted in M1 macrophages.

Excess succinate may also be generated through the 'GABA shunt', in which glutamine is metabolised into glutamate, GABA, succinic semialdehyde and subsequently succinate, bypassing the TCA cycle [55]. As mentioned earlier, excess succinate production can substantially impact inflammatory and metabolic gene expression through the regulation of HIF1α, potentiating inflammation [72]. Glutaminolysis has also been implicated in M2 polarisation through different mechanisms, such as contributing to the production of α-ketoglutarate, which is essential for M2-mediated OXPHOS and FAO, driving epigenetic reprogramming of M2-specific genes [73], and competing with succinate to stabilise HIF1α [74].

There is a large body of evidence that links tryptophan (Trp) metabolism through the kynurenine pathway with peripheral tolerance mechanisms. It has been shown that during inflammation, indoleamine 2,3-dioxygenase (IDO) 1 (IDO1), the first and rate-limiting step in the pathway, is overexpressed. IDO overexpression drives local depletion of Trp, as well as the production of bioactive metabolites, which, through the activation of the stress sensor general control nonderepressible 2 (GCN2) or interactions with specific receptors, can regulate cell division and skew cytokine production towards an anti-inflammatory phenotype [75]. IDO ablation, on the other hand, promotes a proinflammatory profile on immune cells [76–78].

It is widely recognised that arginine is a very important amino acid in CVDs, acting as the substrate for the formation of nitric oxide, which is a key signalling molecule that regulates vascular tone and blood pressure [79]. Hence, arginine has also been implicated in immunity, driving different macrophage phenotypes depending on which pathway the amino acid is engaged in. Under proinflammatory stimuli, such as LPS, TNF or IFNγ, inducible nitric oxide synthase (iNOS) is overexpressed in macrophages, which in turn drives arginine metabolism towards the classic production of NO and citrulline. In macrophages, NO can prevent M1 to M2 repolarisation by interfering with the mitochondrial electron transport chain [80]. Instead of iNOS, M2 macrophages overexpress ARG1, which leads to the formation of urea and ornithine, which is then further metabolised to polyamines and proline. By modulating proline or NO production, ARG1-mediated arginine metabolism has been proposed to be an important mechanism regulating fibrosis and resistance to infection by intracellular mycobacteria [32].

### The metabolic profiles of T cells

A basic T-cell response involves rapid proliferation and the production of effector molecules. In the presence of cognate antigens that are presented by MHC molecules, T cells rapidly shift from a naïve to an activated state, which demands quick energy production through glycolysis [31]. The metabolic profile of activated T cells is characterized by a shift from OXPHOS, which is described as the central energy producing pathway of resting T cells [81]. Compared with macrophages, naïve T cells exhibit lower metabolic rates and low energy demands for their survival.

It has been shown that upon activation, the energy produced via OXPHOS is not sufficient for the new requirements of the T-cell. Activation raises not only energy needs but also the need for intermediate metabolic precursors that can be obtained through the up-regulation of glycolysis. In this context, activated T cells are characterised by increased uptake of glucose and the up-regulation of different glycolytic enzymes, which increase pyruvate and lactate production intracellularly [82]. Through regeneration of the redox cofactor NAD+ and the maintenance of favourable AMP/ATP ratios, glycolysis ensures an abundance of metabolic intermediates for the synthesis of lipids, proteins, and nucleic acids and provides the means for maintaining a stable redox balance [83]. Notably, glycolysis can also directly regulate the secretion of cytokines, for example the enzyme glyceraldehyde 3-phosphate dehydrogenase (GAPDH) can bind to AU-rich elements within the 3ʹ UTR of IFNγ mRNA and influence its expression [84]. Glycolysis has also been linked to the protection of T cells against apoptosis in the context of limited glucose availability [85].

In addition to enhanced glycolysis, T-cell activation also triggers the PPP pathway. This pathway provides precursors for the synthesis of nucleotides and aromatic amino acids, as well as NADPH, which is involved in the maintenance of reduced glutathione and supports lipid synthesis [86,87]. Similar to what happens in macrophages, increased glycolysis can also lead to enhanced HBP, the generation of UDP-GlcNAc and post-translational modifications of essential proteins for T cells [88].

Although glucose is considered the most critical nutrient in T cells, experiments deleting glutamine transporters provided strong evidence that this amino acid may also be essential for T-cell activation [89,90]. In addition to its role as a building block for the TCA cycle, glutamine provides a substrate for UDP-GlcNAc synthesis in T cells [88]. It has been shown that at key stages of T-cell development and activation, glucose and glutamine metabolism occur through the HBP to support dynamics intracellular protein O-GlcNAcylation [88].

After activation, CD8+ T cells proliferate and differentiate into cytotoxic effector cells, whereas CD4+ T cells, depending on the microenvironmental context, can be differentiated into distinct effector T (Teff) cell lineages, including Th1, Th2, Th17, T follicular helper, or regulatory cells (Tregs) [91]. Compared with other Teff cells, Tregs exhibit a peculiar metabolic profile. It has been shown that activation of the mTORC pathway favours the differentiation of Teff cells and suppresses Treg generation [92]. Genetic ablation of the *mTOR* gene in T cells promotes Treg proliferation but not that of Th1, Th2, or Th17 cells [93]. In line with these data, it has been shown that rapamycin-mediated suppression of mTOR can stimulate the development of FoxP3+ Tregs, even in the context of Th17-polarising conditions *in vitro* [94]. We now know that the metabolic profiles of Teff cells are preferentially glycolytic due to mTOR activation, while in Tregs, energy is generated mainly through FAO and OXPHOS [95]. Interestingly, whether mTORC1 or mTORC2 is selectively triggered has been associated with skewed Th1 or Th2 differentiation, respectively [96]. In the context of chronic infections and cancer, it has been shown that many energy metabolism genes become down-regulated in T cells due to exhaustion or anergy. Thus, it has been shown that the restriction of leucine or glucose during T-cell activation can lead to an anergic phenotype [97].

## Metabolic dysregulation in atherosclerosis

Metabolic disorders are a major concern in modern society, and diabetes and obesity are considered independent risk factors for CVD [98,99]. Metabolic syndrome (MetS), which is a term used to define the clustering of at least three major CVD risk factors, has been associated with proinflammatory and prothrombotic states that increase cardiovascular risk beyond that of its individual components, especially in women [100].

Similar immune pathways associated with the acceleration of atherosclerotic plaque formation have been identified in other tissues, for example adipose tissue, the liver, and the pancreas, in the context of metabolic disorders, such as obesity- and MetS-related diseases. Thus, altered numbers and skewed polarisation of leucocytes have been observed in individuals exposed to metabolic disturbances [101].

Recent findings highlighted a substantial impact of MetS-related diseases on ‘nonmetabolic-related’ immune processes in these individuals, such as impaired host defence and immunity against tumours [102]. Notably, obesity has been negatively associated with increased rates of vaccine failure and infection complications [103], raising concern about strategies for the global fight against pandemics, such as COVID-19 [104].

Metabolic dysfunction induced by high-fat diet feeding has been shown to increase inflammation in different tissues, in addition to the aorta in animal models [105]. High-fat diet feeding can influence the immune system at very early stages and skew leucocyte progenitor ratios and myeloid and lymphoid cell development [106]. A growing number of studies indicate that the microenvironmental and cellular metabolism can shape the function and differentiation of immune cells [107]. In this context, activation, proliferation, migration, phagocytosis, and cytokine release can be regulated by intrinsic metabolism [108], which are intracellular metabolic alterations, and/or extrinsic metabolism, which is the availability of nutrients and signalling through specific metabolite-sensing receptors [109].

**Figure 1:**
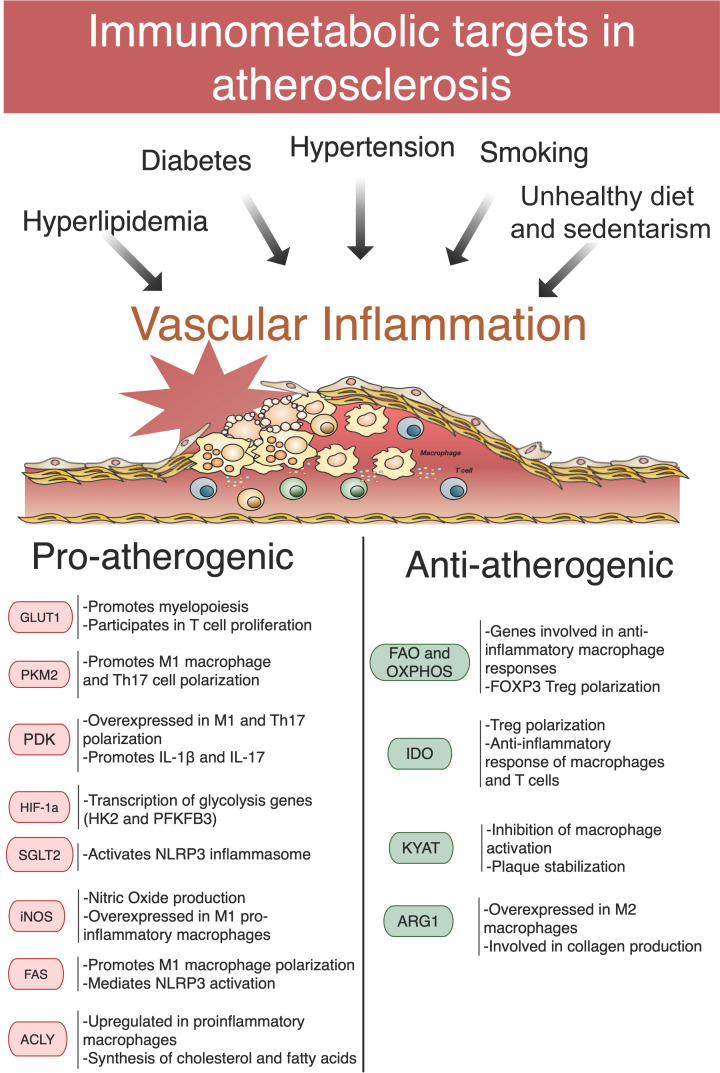
Immunometabolic targets in atherosclerosis Cardiometabolic risk factors such as hyperlipidaemia, diabetes, hypertension, smoking, unhealthy diet, and sedentarism contribute to inflammation in the vascular wall, fibrous cap formation, and eventually rupture of the atherosclerotic plaque. Alterations in metabolic enzymes carry the potential to influence plaque progression (pro-atherogenic) or stabilisation (anti-inflammatory), becoming potential targets for immunometabolic modulation in atherosclerosis. Bottom panel highlights major mechanisms associated with different metabolic enzymes. Abbreviations: ARG1, arginase 1; FAS, fatty acid synthase; GLUT1, glucose transporter 1; HIF-1a, hypoxia inducible factor 1α; KYAT, kynurenine oxoglutarate transaminase; SGLT2, sodium-glucose cotransporter-2.

Recent studies have revealed a complex metabolic environment within atherosclerotic plaques [110,111]. Moreover, it has been suggested that unstable plaques exhibit increased glycolysis, elevated amino acid utilisation, and decreased FAO compared with stable plaques [110]. Considering that atherosclerotic plaques are very complex tissues composed of lipids (cholesterol, cholesteryl esters, and phospholipids), a broad range of inflammatory cells (especially monocytes/macrophages and lymphocytes), SMCs (contractile and transdifferentiated), and several fibrous elements, including connective tissue and extracellular matrix proteins (e.g., collagen, proteoglycans, and fibronectin elastic fibres), the metabolic traits of vascular and immune cells, as well as the accumulation of extracellular metabolites, could help explain different plaque phenotypes (Figure 1).

## Evidence of immunometabolic responses in the atherosclerotic plaque

### Glucose metabolism in macrophages and T cells in atherosclerosis

Early studies using 18-fluorodeoxyglucose (18FDG) positron emission tomography (PET) revealed that both human and mouse atherosclerotic plaques exhibit higher glucose uptake than healthy vessels [112]. Imaging studies showed that macrophages in plaques overexpress glycolytic enzymes and have increased metabolites derived from glycolysis and the PPP, such as citrate, fumarate and succinate, similar to what is observed in activated leucocytes [113].

Experimental research using *ApoE*^*−/*−^ mice revealed that increased glucose uptake in atherosclerotic plaques was linked to a hypermetabolic state and increased *Glut1* expression in haematopoietic cells [114]. Bone marrow transplantation from *Glut1*-deficient mice into irradiated *ApoE^*−/*−^* mice showed that *Glut1* deficiency inhibited myelopoiesis, decreased glucose uptake in the plaque, and slowed atherosclerosis progression [114].

Several studies have provided evidence that CD4 T-cell fate can be modulated by both intrinsic and extrinsic metabolic alterations. Naïve T cells that enter the plaque can be activated and deleteriously or protectively influence atherogenesis [6]. In the absence of antigens, naïve T cells remain quiescent and maintain basal expression of *Glut1* and glucose uptake [115]. However, in the absence of sufficient glucose, T cells fail to proliferate, and they undergo apoptosis [116]. Interestingly, a recent study suggested that subsets of CD4 T cells from atherosclerotic mice exhibited impaired glucose metabolism, as evidenced by decreases in *Glut1* expression and FAO oxidation-related genes [117]. It has also been suggested that T-cell receptor signalling in T cells in the lipid-rich atherosclerotic plaque environment can modulate T-cell metabolism and induce functional changes that lead to activation, proliferation and exhaustion [117].

In human atherosclerosis, glycolytic regulation of macrophage function seems to be crucial. Previous research has shown that monocytes and macrophages from patients with atherosclerosis exhibit increased glycolytic flux [118]. This increased glycolysis promotes PKM2 dimerisation and nuclear translocation, leading to increases in IL-6 and IL-1β expression. Inhibition of glycolysis or the induction of PKM2 tetramerisation corrects the proinflammatory phenotype of macrophages from patients with coronary artery disease [118]. Additional *ex vivo* studies with human atherosclerotic plaques have shown that oxidised LDL (oxLDL) and hypoxia increase glycolysis and promote a proinflammatory phenotype in macrophages [119,120]. Thus, increased glucose uptake in atherosclerotic plaques could be a consequence of macrophage adaptation to hypoxia and the plaque metabolic microenvironment. However, it has been recently revealed that macrophage activation by oxLDL can increase glycolysis independent of HIF1α activation but still regulate the expression of glycolytic enzymes such as hexokinase 2 (HK2) and 6-phosphofructo-2 kinase/fructose-2,6-biphosphatase 3 (PFKFB3) [119,120].

T-cell polarisation into either Treg or Th17 lineages depends on the combination of cytokines, as well as metabolic substrate availability. Th17 cells resemble M1 macrophages and overexpress glycolytic enzymes such as PKM2 or PDK1. While the inhibition of glycolysis in Th17 cells has been linked to preferential expansion of Tregs [121], the inhibition of glycolytic enzymes in atherosclerosis could have potential implications for both M1 macrophages and Th17 cells. In this context, the inhibition of PKM2 tetramerisation in CD4 T cells has been demonstrated to reduce hyperhomocysteinemia-accelerated atherosclerosis [122].

It has been previously established that hyperglycaemia, which is associated with type 1 or type 2 diabetes mellitus, is an independent risk factor for atherosclerotic CVD [123,124]. However, the explanation about the mechanisms that could help trigger or accelerate disease remain incomplete. Macrophages from patients with diabetes show an inflammatory phenotype [125]. It has been shown that hyperglycaemia promotes myelopoiesis and exacerbates atherosclerotic lesions [126], and that lowering glucose levels can correct this phenotype [125].

It has been suggested that glucose lowering could be an effective strategy to decrease atherosclerotic risk in diabetic patients. Although glycaemic control reduces major cardiovascular events in type 1 diabetes patients [127], several studies conducted on type 2 diabetes (T2D) patients showed that lowering glucose levels did not reduce cardiovascular risk [128]. Thus, it has been speculated that other cardiovascular risk factors, such as dyslipidaemia, obesity and hypertension, which are also often associated with T2D, could mask the effect of the conventional treatment of glucose lowering [129].

The recent use of new classes of antidiabetic drugs, such as sodium glucose cotransporter-2 (SLG2) inhibitors and the incretin hormone glucagon-like peptide 1 (GLP1) agonists, seems to have contradicted the previous dogma by showing glucose lowering associated with a significant reduction in CVD risk [130]. Although it remains unclear why these drugs have cardioprotective effects while other drugs that also reduce glycaemia do not, recent studies suggest these new drugs can also influence immunity. It as been proposed that SLG2 inhibition reduces atherosclerosis in *ApoE*^−/−^ mice through regulation of the NLRP3 inflammasome expression in macrophages [131]. Interestingly, it has been shown that a subset of T cells in the gut can modulate systemic metabolism and contribute to CVD by limiting the bioavailability of GLP1 [132].

Still in the context of potential explanations for why lowering glucose levels *per se* does not influence CVD risk, it has been proposed that hyperglycaemia can induce ‘trained immunity’ in myeloid precursors and differentiated cells. Trained immunity is characterised by the ‘long-term’ epigenetic reprogramming of innate immune cells mediated by exogenous or endogenous insults, conferring ‘memory’ to these cells even when they return to a non-activated state [133]. This phenomenon has, for example, been identified in circulating leucocytes from T2D patients [134,135]. It has been shown that in diabetic mice, high glucose levels induce epigenetic modifications in bone marrow progenitors that persist until after these cells differentiate into macrophages. Interestingly, the transplantation of haematopoietic stem cells from mice with diabetes to euglycaemic recipient mice was shown to be sufficient to accelerate atherosclerosis [136], indicating that metabolic imprints on immune cell precursors could be sufficient to jeopardise some therapeutic approaches.

### Lipid metabolism in macrophages and T cells in atherosclerosis

Atherosclerotic plaques are especially rich in lipids. The uptake of modified lipids, such as oxLDL, via scavenger receptors on macrophages is a key feature in the formation of foam cells. Subsequent accumulation leads to the formation of fatty streaks and advanced atherosclerotic lesions. LDL modifications can alter macrophage metabolic activity [137]. *In vitro* and *in vivo* studies have shown that oxLDL can up-regulate glycolysis, inflammation and oxidative stress in macrophages [138,139]. Interestingly, myeloid cells from *LDLr*^−/−^ mice fed a high-fat diet exhibited signs of long-lasting epigenetic changes and metabolic reprogramming that are characteristic of trained immunity [138].

Alterations in cholesterol metabolism have been associated with numerous consequences to immunity. For example, in innate immune cells, changes in intracellular cholesterol can lead not only to foam cell formation but also inflammasome activation on macrophages, and neutrophil extracellular traps (NETs)-induced cell death or NETosis by neutrophils [140]. It has been shown that enrichment of cholesterol in lipid rafts in human pluripotent stem cells promotes proliferation and mobilisation of bone marrow cells, leading to leucocytosis [141–143]. In this context, liver X receptor (LXR)-mediated signalling, and the regulation cholesterol efflux, including the transcription-regulation of the ATP-binding cassette transporters ABCA1 and ABCG1, have been proposed as major regulators of cholesterol-driven exacerbation of immune responses [144–147].

As mentioned earlier, FAS and FAO play important roles in macrophage polarisation [148,149]. In this context, the multicomplex enzyme FASN has been shown to play an important role in M1 macrophage polarisation [150]. Considering the proinflammatory nature of M1 macrophages, specific deletion of *Fasn* in macrophages was shown to reduce atherosclerotic plaque formation and foam cell formation in *ApoE*^*−/*−^ mice [151]. It has been shown that hypoxia, which is frequently found in advanced atherosclerotic plaques, enhances FAS, suppresses FAO, and promotes triglyceride-laden macrophage formation [152]. Interestingly, based on single-cell analysis, two recent studies suggested that foam cells do not express inflammatory cytokines and that instead, these cells could help suppress inflammatory responses in the plaque [40,41].

It has been recently reported that oxidised 1-palmitoyl-2-arachidonyl-sn-glycero-3-phosphorylcholine (oxPAPC), which is considered a proatherogenic modified fatty acid, potentiates the effects of LPS on macrophage metabolism, inducing a hypermetabolic state on these cells that accelerates atherosclerosis. Surprisingly, oxPAPC-triggered cells resembled a mixed metabolic profile of M1 and M2 macrophages, including increased glycolysis and OXPHOS, which was paralleled with large amounts of IL-1β production, a characteristic of only M1 macrophages [153]. Although these data initially sounded confusing, raising questions to which extent metabolic skewing can govern macrophage polarisation in the atherosclerotic plaque, they highlight how complex macrophage repertoires can be in chronic inflammatory processes. Moreover, this finding may help explain why targeting inflammation to prevent CVD remains challenging.

It has been shown recently that the expression of the surface marker TREM2 (Triggering-receptor-expressed on myeloid cells 2) on macrophages (TREM2hi) in the intima was associated with the expression of genes linked to lipid metabolism and cholesterol efflux [41]; TREM-2 that is also highly expressed in adipose tissue-associated macrophages (ATMs). In this context, it has been shown that mice lacking TREM2 do not form ATM populations during obesity, and glucose homoeostasis in adipose tissue is influenced [154]. Whether TREM2+ macrophages link to lipid overload and lipotoxicity play a role in the progression or regression of atherosclerosis warrants deeper investigations.

ACLY, which is involved in fatty acid and cholesterol biosynthesis by influencing acetyl-CoA formation from citrate, has been recently shown to be up-regulated in inflammatory macrophages in human atherosclerotic plaques [155]. Hence, hypercholesterolaemic mice with ACLY deficiency in myeloid cells developed macrophages with deregulated fatty acid and cholesterol synthesis and a more stable plaque phenotype [156]. In line with these data, bempedoic acid, an ACLY inhibitor that is used to reduce cholesterol synthesis and treat dyslipidaemia, was shown to attenuate atherosclerosis in *ApoE^−/−^* mice and *LDLr^−/−^* mice [157]. Recent clinical trials have shown that bempedoic acid significantly lowers LDL as a monotherapy or combination therapy and could have add-on effects with statin therapy in statin-intolerant patients [155]. The fact that ACLY inhibition influences vascular inflammation and therogenesis in murine models suggests that targeting this enzyme could be a promising therapeutic strategy to combat CVDs.

Tregs are unable to develop in the absence of fatty acids or when FAO is inhibited. On the other hand, palmitate exposure can selectively induce the apoptosis in Teff cells, suggesting that the availability of certain lipids can skew the Treg/Teff ratio. In this context, it has been shown that *Foxp3* expression can regulate the expression of enzymes associated with FAO and mitochondrial OXPHOS [158]. *FOXP3* expression can selectively impair the survival of cells exposed to saturated fatty acids and in environments with low glucose and high lactate, such as atherosclerotic plaques or the tumour microenvironment [159].

The mechanisms of fatty acid biosynthesis and cholesterol synthesis are strongly up-regulated in activated T cells [81]. Cholesterol and cholesterol derivates can shape plasma membrane fluidity and participate in the dynamics of lipid rafts, thereby changing the colocalisation of crucial receptors, including the immunological synapse form during TCR recognition of epitope-MHC-II on antigen presenting cells [160]. Cholesterol levels in proliferating T cells are maintained in part through the opposing transcriptional activities of sterol regulatory element-binding protein 2 (SREBP2) that is up-regulated, and the LXR that is down-regulated [145].

It has been shown that intracellular cholesterol homoeostasis is crucial for maintaining Treg stability and function [161]. However, a substantial accumulation of intracellular cholesterol, which occurs in atherosclerosis, may disrupt homoeostasis and promote disease progression through the conversion of Tregs into T effector cells [162]. Studies on *Apoe*^*−/*−^ mice showed that high-fat diet induced an increase in esterified cholesterol in Tregs. Treatment of these mice with apolipoprotein A1 (ApoA1) increased the expression of the cholesterol efflux transporter ABCA1 in Tregs and normalised the cholesterol content in these cells. Of interest, ApoA1 treatment inhibited the conversion of Tregs into proatherogenic T follicular cells and reduced atherosclerosis [163]. Hence, it has been hypothesised that the accumulation of intracellular cholesterol can affect membrane lipid rafts in which IL-2 receptors are enriched, thereby abolishing IL-2 signalling and Treg function and homoeostasis [164].

### Amino acid metabolism in macrophages and T cells in atherosclerosis

The metabolism of amino acids has also been implicated in the atherogenic responses of macrophages and T cells [165]. Metabolomics analysis of plasma from *Ldlr^−/^^*−*^* mice showed that the levels of the amino acids glycine, glutamine and valine, in parallel with other metabolites such as lactate and citrate, were increased in late stages of atherosclerosis [166]. The same study indicated that some inflammation driven changes in amino acid metabolism were associated with the inhibition of atherosclerosis [166].

One of the most studied amino acids in the context of inflammation is glutamine. In *in vitro* studies, using murine macrophages, it has been suggested that glutamine can promote pro-atherosclerotic responses [167]; this study showed that macrophages from *Apoe^*−/−*^* mice supplemented with excess glutamine exhibit increased triglyceride biosynthesis due to the activation of SREBP1, and that peritoneal macrophages presented increased ROS production [167]. However, the definitive proof on whether glutamine supplementation could influence atherosclerosis was not evaluated, allowing the possibility of doubt that this amino acid can induce deleterious responses to the vascular wall.

Glutamine plays a key role in cell metabolism by feeding the TCA cycle, which involves the formation of glutamate and its conversion into α-ketoglutarate [52]. In macrophages subjected to trained immunity, marked increases in succinate and fumarate levels, which have been associated with the inhibition of the histone demethylase, Lysine-specific demethylase 5A (KDM5) and a proinflammatory phenotype, have been observed. Providing macrophages with α-ketoglutarate, the substrate for KDM5, increased KDM5 availability and suppressed the proinflammatory phenotype [168]. In this context, increased glutaminolysis and the promotion of α-ketoglutarate formation have been identified as important anti-inflammatory mechanisms that regulate O-GlcNAcylation and inflammation in human adipose tissue [169].

Upon activation, immune cells go through dramatic changes in metabolism to fulfil the bioenergetic, biosynthetic and redox demands of proliferation and differentiation, including increased glutamine metabolism. Increased glutamine metabolism can lead to important antioxidant and anti-inflammatory effects such as the induction the expression of Haem oxygenase 1, heat shock proteins and glutathione; the latter that plays an essential role in controlling redox balance and T cell fate [170], by exerting a strong antioxidative role and protecting cell against stress-induced damage [171]. However, excessive shunting of glutamine to the TCA cycle has been shown to lead to aberrant proinflammatory responses. In these instances, therapeutic targeting of the enzymes involved in glutaminolysis has shown promise in preclinical models investigating angiogenesis [172]. Whether targeting glutaminolysis is relevant in the context atherosclerosis will require further investigations.

L-Arginine metabolism and its by-product nitric oxide have been proven to be vitally important for the early stages of atherosclerosis [173], such as protecting against endothelial dysfunction [174]. However, eNOS overexpression, which can be seen at later stages of disease, lead to the overproduction of NO and has been considered a mechanism of disease acceleration [175]. As competing enzymes for L-arginine metabolism, the two enzymes involved in the degradation of this amino acid, iNOS and ARG1 are known regulators of macrophage responses [176]. In this context, it has been shown that both human and murine atherosclerotic plaques express ARG1 and that its overexpression promotes atherosclerotic plaque stabilisation [165,177].

Tryptophan is another essential amino acid that has been proven to influence atherosclerosis. Genetic and pharmacological inhibition of IDO-1 led to substantial increase in vascular inflammation and atherosclerosis in *Apoe*^*−/*−^ mice [178,179]. In line with these data, IDO1 induction has been linked to atheroprotection and increased plaque stability [180,181]. It has been proposed that IDO-1 expression affects atherosclerosis via multiple mechanisms. Eicosapentaenoic acid administration stimulates IDO-1 expression and reduces vascular inflammation and atherosclerosis in *Ldlr*^−/−^ mice, possibly by decreasing the numbers of macrophages, dendritic and T cells [182]. Tregs participate in atherosclerosis by counteracting pro-inflammatory signals and promoting plaque stabilisation [183]. Tregs can also influence inflammation by inducing IDO-1 expression in antigen-presenting cells [180], which can create positive feed–forward loop to increase their numbers. Although an attractive target, IDO-1 stimulation could, however, lead to undesired effects, such as defective immunity and increased susceptibility to infection [184].

Not only IDO1 but also other enzymes involved in tryptophan degradation have been associated with regulation of inflammation, and a deviation in the kynurenine pathway, due to the reduction in the expression of kynurenine oxoglutarate aminotransferases (*KYAT*s), has been associated with an increased probability of developing symptomatic unstable atherosclerotic disease. The same study suggested that signalling through aryl hydrocarbon receptor (AhR), mediated through the bioproduct of the KYATs kynurenic acid (KynA), could be a key mechanism regulating vascular inflammation [185].

A deleterious role of this pathway has also been reported in the context of atherosclerosis. It has been shown that human atherosclerotic lesions can exhibit increased levels of KynA, which was associated with an unstable plaque phenotype and the recurrence of myocardial infarction in patients with coronary artery disease [186]. Hence, the same group showed that endothelial cells (ECs) but not inflammatory cells from mice with myocardial infarction were the main producers of KynA. Mice with specific deletion of IDO1 in ECs showed improvements in cardiac function, as well as cardiomyocyte contractility, and a reduction in adverse ventricular remodelling [187].

## The metabolic regulation of vascular cell inflammatory responses

To maintain vascular homoeostasis, ECs mainly use glycolysis and other glycolytic pathways for ATP generation, such as the HBP and the PPP, rather than OXPHOS [188]. It has been suggested that the low mitochondrial volume in ECs in comparison with other cell types could explain this metabolic preference in the healthy state [189]. Mitochondria seems to play a pivotal role in endothelial function, as it has been shown that altered mitochondrial dynamics can drive the development of endothelial dysfunction in some metabolic diseases, such as T2D, and atherosclerosis [190,191].

The full understanding of the molecular mechanisms linking mitochondrial and EC dysfunction has not been completely elucidated. ECs are more sensitive to ROS damage than other vascular cells, such as SMCs [192]. ECs in *ApoE^−/−^* mice and human atherosclerotic plaques exhibit mitochondrial DNA (mtDNA) damage due to ROS, which correlated with the extent of atherosclerosis [193]. In addition, it has been revealed that inherited mtDNA damage mutations in SMCs could initiate vascular damage and promote atherosclerosis [194]. However, the relationship between atherosclerosis and mitochondrial mutations in ECs remains unexplored.

Emerging evidence indicates that epigenetic mechanisms may be implicated in the endothelial dysfunction that precedes vascular complications in T2D. The concept of ‘glycaemic memory’ in ECs refers to constant exposure to a hyperglycaemic environment that is imprinted on the ECs of diabetic patients whose glycaemic control was achieved very early, consequently leading to endothelial dysfunction and vascular complications [195]. Interestingly, ECs metabolise excess glucose into sorbitol via the polyol pathway, which increases ROS and advanced glycation end product (AGE) formation, inducing glycation of respiratory chain proteins and DNA damage [196]. Genome-wide sequencing studies on ECs exposed to a high level of glucose revealed that hyperglycaemia could induce epigenetic changes in the vascular endothelium that were associated with atherosclerosis development, thereby providing another link between diabetes and atherosclerosis pathogenesis [196].

SMCs control vascular tone and diameter through contraction and relaxation. Specially in arteries, SMCs may be not terminally differentiated and exhibit phenotypic plasticity that is regulated by environmental cues [197]. In atherosclerosis, SMCs are stimulated to dedifferentiate from a quiescent contractile phenotype to a synthetic phenotype. This change is accompanied by decreases in the expression of SMC-specific contractile genes and increases in proinflammatory genes that induce proliferation and migration [198].

It has been described that SMCs adapt their metabolism to this phenotypic switch. Thus, contractile SMCs mainly rely on OXPHOS and FAO as sources of acetyl-CoA, while synthetic SMCs preferentially use glycolysis to proliferate and migrate [199]. It has been demonstrated that SMC polarisation towards a synthetic phenotype requires metabolic reprogramming characterised by an increased glycolytic rate and the stabilisation of HIF1α [200]. Hence, hypoxia and increased lactate production could be important factors that promote the synthetic phenotype [201]. In addition to lactate, it has been recently shown that glutamine also promotes a synthetic phenotype through the down-regulation of miR-143, which affects the proliferation and migration of SMCs [202].

Stable atherosclerotic plaques are characterised by a dense fibrous cap that can be composed by myofibroblasts that transdifferentiate from SMCs. Recent data from single-cell mRNA sequencing have identified that the transdifferentiation of SMCs via endothelial-to-mesenchymal transition (endoMT) induced by IL-1β and TGFβ is dependent of glycolysis [203].

## Conclusions

**Figure 2:**
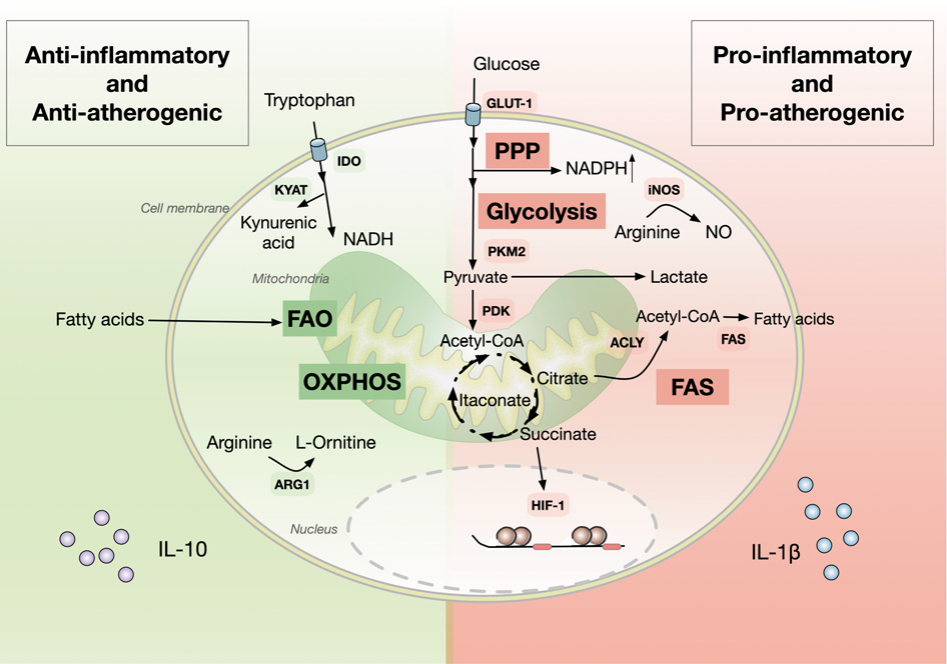
Major immunometabolic pathways involved vascular inflammation and atherosclerosis Pro-inflammatory/pro-atherogenic and anti-inflammatory/anti-atherogenic immune responses are characterised by distinct metabolic traits. Whereas OXPHOS and FAO prevail in anti-nflammatory/anti-atherogenic immune cells (e.g., M2 macrophages and Tregs), glycolysis, PPP, and fatty acid synthesis (FAS) are characteristic in in pro-inflammatory/pro-atherogenic immune cells (e.g., M1 macrophages and T effector cells). Abbreviations: ARG1, arginase 1; FAS, fatty acid synthase; GLUT1, glucose transporter 1; HIF-1a, hypoxia inducible factor 1α; IDO, indoleamine 2,3-dioxygenase; iNOS, inducible nitric oxidase; SGLT2, sodium-glucose cotransporter-2.

In this review, we summarised several relevant findings of the metabolic regulation of immune cells (Figure 2), as well as their potential consequences in atherosclerosis. Increased energy metabolism characterised by high glycolysis, hypoxia, a truncated TCA cycle, fatty acid synthesis and defective amino acid metabolism are associated with inflammation and lipid accumulation in the atherosclerotic plaque. On the other hand, high OXPHOS and FAO are associated with decreased inflammation and potentially with limited atherosclerosis progression.

Although the response to specific metabolites or skewing towards a metabolic route has been extensively studied in simple systems *in vitro*, knowledge of the effects of metabolic alterations on pathophysiological processes *in vivo* remains limited. In this context, in the field of immunometabolism, there is a need for a better understanding of the metabolic adaptations to complex microenvironments. Increasing the number of studies characterising disease tissues using new state-of-the-art technologies, including single-cell transcriptomics and proteomics analyses, combined with classic tissue pathology characterisation, as well as tissue metabolomics, will help researchers better understand the complexity of different signals and metabolic signatures that influence immune and vascular cells. In this context, advancing emerging techniques, such as Matrix-assisted laser desorption/ionisation-mass spectrometry (MALDI-MS) for single-cell and subcellular analysis [204,205], carry the potential to revolutionise our views of complex inflammatory processes, in the atherosclerotic plaque and beyond. The full potential of multiomics analyses to decipher immunometabolism is discussed, in depth, in the excellent review by Artyomov and Van den Bossche (2020) [206].

While many studies are still needed to clarify the role of immune and vascular cell metabolism in atherosclerosis, the studies cited here indicate that targeting immunometabolism to repolarise immune cells towards an anti-inflammatory phenotype appears as a promising therapeutic strategy to reduce atherosclerosis. A major limitation of interfering with the metabolism of immune cells could be that systemic delivery of drugs can affect other cells and organs, possibly generating deleterious off-target effects. The identification of more selective drugs that can target unique characteristics of pro- or anti-inflammatory cells would be a breakthrough for future disease treatment. In this context, targeting enzyme isoforms that are predominantly expressed in proinflammatory cells and have low expression in anti-inflammatory or stromal cells could have good specificity and reduce unwanted side effects.

An interesting point of reflection raised by this review is the fact that metabolic changes can affect immune cells precursors in the bone marrow. This epigenetic memory imprinted by metabolic changes may represent a risk of continuous skewing of inflammatory cells. Being able to reverse deleterious epigenetic signatures in progenitor cells could be an efficient way to create stable anti-inflammatory responses.

In summary, there is accumulating evidence that immunometabolism is affected by systemic pro-atherogenic factors in the blood and bone marrow niche, as well as within the atherosclerotic plaque environment. Further elucidation of the metabolic mechanisms governing vascular inflammation and atherogenesis carries a high potential to provide novel pharmacological targets to combat CVDs.

## Open Access

Open access for this article was enabled by the participation of Karolinska Institute in an all-inclusive *Read & Publish* agreement with Portland Press and the Biochemical Society.

## Abbreviations

ACLY: ATP-citrate lyase
ApoA1: apolipoprotein A1
ATM: adipose tissue-associated macrophage
CoA: coenzyme A
CVD: cardiovascular disease
EC: endothelial cell
FAO: fatty acid oxidation
GLP1: glucagon-like peptide 1
HBP: hexosamine biosynthesis pathway
HIF1α: hypoxia-inducible factor-1α
IDO: indoleamine 2,3-dioxygenase
IFNγ: interferon γ
iNOS: inducible nitric oxide synthase
KDM5: lysine-specific demethylase 5A
KYAT: kynurenine oxoglutarate transaminase
KynA: kynurenic acid
LDL: low-density lipoprotein
LXR: liver X receptor
MetS: metabolic syndrome
MHC: major histocompatibility complex
mtDNA: mitochondrial DNA
NADPH: nicotinamide adenine dinucleotide phosphate
oxLDL: oxidised LDL
oxPAPC: oxidised 1-palmitoyl-2-arachidonyl-sn-glycero-3-phosphorylcholine
OXPHOS: oxidative phosphorylation
O-GlcNAc: O-linked B-N-acetylglucosamine
PDK1: pyruvate dehydrogenase kinase 1
PKM2: pyruvate kinase M2
PPP: pentose phosphate pathway
ROS: reactive oxygen species
SLG2: sodium glucose cotransporter-2
SMC: smooth muscle cell
Teff: effector T
Th: T helper
Treg: regulatory T cell
T2D: type 2 diabetes
UDP-GlcNAc: uridine diphosphate N-acetylglucosamine
